# Abnormal Gastroscopy Findings Were Related to Lower Meridian Energy

**DOI:** 10.1155/2011/878391

**Published:** 2010-11-02

**Authors:** Sheng-Miauh Huang, Li-Yin Chien, Chun-Chao Chang, Ping-Ho Chen, Chen-Jei Tai

**Affiliations:** ^1^School of Nursing, National Yang-Ming University, Taipei 112, Taiwan; ^2^Institute of Clinical and Community Health Nursing, National Yang-Ming University, Taipei 112, Taiwan; ^3^Department of Gastroenterology, Taipei Medical University Hospital, Taipei 110, Taiwan; ^4^Department of Traditional Chinese Medicine, Taipei Medical University Hospital, Taipei 110, Taiwan; ^5^Department of Medicine, Taipei Medical University, Taipei 110, Taiwan

## Abstract

According to the theories of Traditional Chinese Medicine (TCM), energy runs through 12 meridians longitudinally up and down the body. The study objectives were to compare the meridian energy between subjects with and without abnormal gastroscopy findings. We applied a cross-sectional and correlational research design. The study included 1,223 participants who had their health examinations at a university hospital in Taipei from 1st August 2005 through 31st August 2007. Meridian energy was examined using a meridian energy analysis device. The gastroscopy was operated by certified gastroenterologists. Participants with abnormal stomach and esophageal findings using gastroscopy had significantly lower mean meridian energy. There were no significant differences in meridian energy between participants with and without abnormal duodenum findings. When all of the meridians were examined individually, participants with abnormal findings in esophagus and stomach had significantly lower meridian energy in each of the meridians. The results of this study demonstrated that structural abnormality in the gastric area was related to lower meridian energy. Whether enhancing meridian energy could improve gastric and esophageal health merits further studies.

## 1. Introduction

According to the theories of Traditional Chinese Medicine (TCM), qi and blood run throughout the body via meridian vessels. The interconnected meridian vessels are composed of 12 main meridians, eight extra meridians, and collateral vessels [1–5]. Qi and blood running through meridians are regarded as meridian energy [6, 7]. Health of an individual is viewed as a function of level and balance of meridian energy (or qi and blood) according to TCM practitioners [1–5]. 

 There are 12 main meridians bilaterally in the body. The left and right meridians are symmetrical to the center of the body and influence each other through the interconnected meridian vessels [1, 2, 4]. The 12 main meridians included three yin meridians and three yang meridians for the hand and the foot (one side). The three yin meridians of the hand, running through the anterior of the upper limbs from the chest to the hands, are the lung meridian, heart meridian, and pericardium meridian. The three yang meridians of the hand, flowing through the posterior of the upper limbs from the hands to the head, are the large intestine meridian, small intestine meridian, and triple energizer meridian. The three yin meridians of the foot, running through the medial side of the lower limbs from the feet to the abdomen and chest, are the spleen meridian, kidney meridian, and liver meridian. The three yang meridians of the foot, flowing from the head through the back downward to the feet, are the stomach meridian, bladder meridian, and gallbladder meridian [1, 2, 8]. 

 It is believed that meridian and collateral vessels connect the bowels, viscera, extremities, and superficial organs and tissues [2–4]. Meridian energy may reflect problems of viscera and bowels, which may be treated by TCM medication and/or acupuncture to modulate the levels and balance of meridian energy. When TCM doctors try to improve patients' health by modulating the balance of qi/blood in the body, they consider the dynamic balance of the whole body and reduce the differences of imbalances, such as yin and yang, left and right sides, upper and lower limbs, and the maximum and minimum [2–5]. 

A systematic review confirmed the existence of meridians, although their full nature has not been revealed [1]. It is generally assumed that electrical conductivity measurements of meridians provide information about meridian energy [9, 10]. In the present study, we used Ryodoraku equipment to measure meridian energy. Many previous studies have used the Ryodoraku to measure energy levels [9–14]. The 24 measured points of the Ryodoraku are located on the twelve main meridians, and therefore the resultant electrodermal measurement is regarded as the meridian energy based on TCM theories [10, 13]. 

 Of all the cancers in Taiwan, gastric carcinoma ranked the fifth in rate of incidence and mortality. Death due to gastric cancer in 2007 was 8.5 per 100,000 people in Taiwan [15], which was higher than that in the United States (3.7/100,000) [16] and lower than that in Japan (male: 34.2/100,000; female: 14.1/100,000) [17]. From the results of gastroscopy, the prevalence of gastritis varied from 47% in the United Kingdom to 60% in Japan with antrum and corpus being the most common lesions [18]. There is no data concerning the prevalence of gastritis in Taiwan. Research has suggested that chronic gastritis, gastric atrophy, and intestinal metaplasia might lead to dysplasia and stomach cancer, especially for those with corpus gastritis [18–21]. Thus early detection and treatment of those stomach problems is important. Gastroscopy is by far the best tool to detect stomach problems [18, 22]. 

 To our knowledge, there have been no studies linking meridian energy to structural problems of organs. The objectives of this study were to compare the meridian energy between subjects with and without abnormal gastroscopy findings.

## 2. Materials and Methods

 The participants were adults who received health checks at a university hospital in Taipei. The health check package included the modern medical checkup offered by the Department of Family Medicine and the meridian energy checkup offered by the Department of Traditional Medicine. The study was approved by the human subjects committee at the hospital (approval number CRC-07-09-02). This was a chart review study and involved no contact with the patients. The patient names were replaced by coded numbers to ensure anonymity after data abstraction completed. Written consent was not required, since patient identifiers were not included in the data.

### 2.1. Study Participants

 There were 1,244 persons who accepted the above-mentioned health check package from 1st August 2005 through 31st August 2007. We excluded people who were younger than 18 years of age, who had cancer, and who were pregnant. As a result, 1,223 people were enrolled. The mean age of the subjects was 47.24 years (SD = 12.27; range, 20–85 years). About 55.8% of the participants were men. More than one-fifth (21.8%) had a chronic disease (Table 1).

 According to the standard health-check procedures, clients were first assessed by a medical doctor regarding their signs, symptoms, and general health status. The clients were asked whether they would like to receive the gastroscopy. In addition, the medical doctors assessed each client's symptoms, when the doctors found signs suggesting gastric problems, they arranged for gastroscopy examinations. Therefore, patients received gastroscopy either by request or by doctor's referral. Of the 1,223 participants, 665 received gastroscopy. All participants received meridian energy checks. 

Of those who received gastroscopy (*n* = 665), 636 (95.6%) had abnormal findings and only 29 (4.4%) had no abnormal findings. We assumed that participants who did not receive gastroscopy were free from abnormal gastroscopy results. The participants were divided into 2 groups, one with (*n* = 636), and the other without abnormal gastroscopy findings (the 29 persons who received gastroscopy without abnormal findings plus 558 persons who did not receive gastroscopy).

### 2.2. Measurements

 The meridian energy was examined by MEAD Me-Pro (Hanja International CO. Ltd, Taoyuan, Taiwan), which yielded electrodermal measurements of the 24 meridian (12 for the left and 12 for the right) and is similar to equipment used in previous studies [11–14]. The design of MEAD is based on the Ryodoraku theory. The machine has two electrodes. One is a metal cylinder that is held in the left hand of the patient. The other electrode is connected to a spring-loaded probe that contains cotton moistened with physiological saline solution; a trained technician applies this electrode to the 24 energy points that lay along the 12 meridians. The measurements are started with very low current, which is gradually increased to a maximum value of 200 *μ*A. Readings of the electrical conductivities of the meridians are directly entered into a computerized system.

Before the study was conducted, 30 people were recruited to examine the stability of the MEAD data. MEAD measurements were done twice for each person, 15 minutes apart. The correlation of the two measurements in the same subjects was high (*r* = 0.88), suggesting that the MEAD measurements were reliable. 

The level of the meridian energy was determined using the MEAD values for each of the 24 meridians. The meridian energy for each meridian ranged from 0 to 200 *μ*A. The overall meridian energy was the average of 24 meridian energy results. The balance of the meridian energy was measured by the values of the upper/lower, left/right, yin/yang, and max/min ratios. The value of the upper/lower ratio was the meridian energy of the upper limbs divided by that of the lower limbs; same concepts were applied to yield the left/right, yin/yang, and max/min ratios. To achieve the appropriate balance for the energy results, a ratio of close to one was preferred. The meridian energy data were transferred directly from the machine to the computer database. 

 Patients were required to take nothing by mouth (NPO) for at least 8 hours before they underwent gastroscopy and meridian energy checkup according to hospital procedures. Before the meridian energy checkup, the participants removed their shoes, socks, metal objects, and cell phones; then they rested for 15 minutes before the measurements started. The technician and patients were both blinded to the gastroscopy results at the time of the MEAD measurements. 

 The gastroscopy was operated by 10 physicians who had undergone training for more than 5 years in the gastrointestinal department and were certified by the Gastroenterological Society of Taiwan. In the gastroscopy reports, the lesion site and findings were recorded.

### 2.3. Data Analysis

 Statistical analyses were performed using the SPSS 15.0 software (SPSS Inc, Chicago, Ill, USA). Individual variables were examined using percentages, means and standard deviations. Group differences were explored using *χ* ^2^ statistics and *t*-tests.

## 3. Results

### 3.1. Gastroscopy Results

 Among all the participants, 636 subjects (52.0%) had at least one problem in esophagus, stomach and duodenum. Abnormal lesions at the antrum (39.4%) and corpus (35.3%) were the most common. The number of people who had two abnormal stomach lesions was 356 (29.1%), and three lesions was 27 (2.2%). The rate of gastritis, ulcers, polypoid, metaplasia, and atrophy were 46.0%, 5.3%, 4.3%, 0.7%, and 0.6%, respectively. Esophagitis was found in 133 (10.9%) participants and hiatus hernia was found in 102 (8.3%) participants. Bulbitis and duodenum ulcer had a rate of 5.8% and 7.3%, respectively.

### 3.2. Overall Meridian Energy

**Figure 1:**
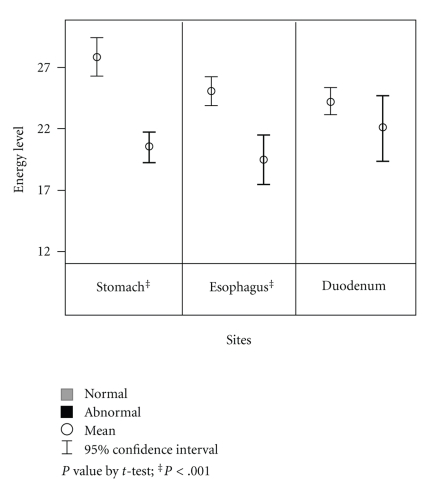
Mean level of meridian energy by sites of gastroscopy findings (normal, abnormal).

 The mean of overall meridian energy was 24.1 (SD = 17.5). Participants with abnormal gastroscopy findings in the stomach and esophagus had significantly lower meridian energy (stomach: 20.53 versus 27.90, mean difference = −7.36, *P* < .001; esophagus: 19.58 versus 25.19, mean difference = −5.61, *P* < .001). There were no significant differences in the meridian energy between participants with and without abnormal duodenum findings (22.16 versus 24.36, mean difference = −2.20, *P* > .05; See Figure 1).

### 3.3. Balance of Meridian Energy

**Figure 2:**
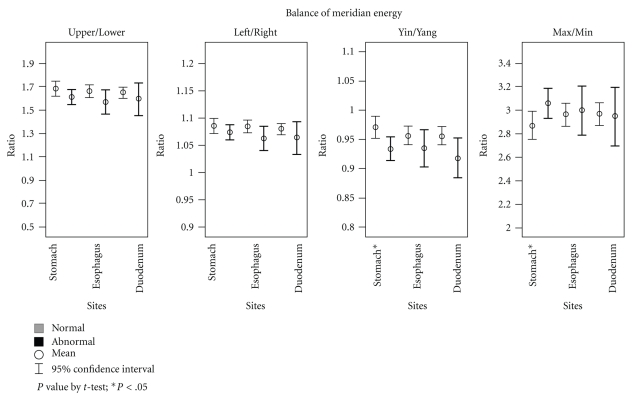
Balance of meridian energy by sites of gastroscopy findings.

In terms of balance of meridian energy, participants with abnormal stomach findings had significantly lower yin/yang ratios (stomach: 0.93 versus 0.97, mean difference = −0.04, *P* < .05) but significantly higher max/min ratios (3.06 versus 2.87, mean difference = 0.19, *P* < .05). There were no significant differences in the upper/lower, left/right, yin/yang, max/min ratios between the participants with and without abnormal findings in the esophagus and duodenum (Figure 2).

### 3.4. Individual Meridian Energy

**Figure 3:**
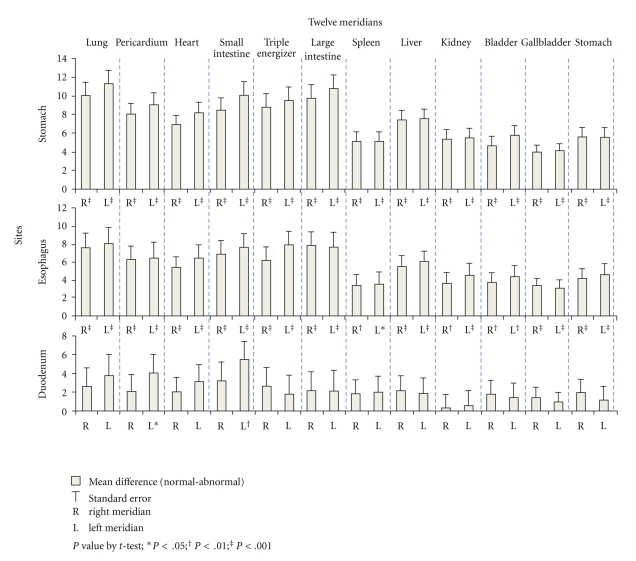
Mean difference of meridian energy between participants without and with abnormal gastroscopy findings.

 When the 24 meridians were examined individually (mean differences between normal and abnormal groups were presented in the Figure 3), participants with abnormal gastroscopy findings in the esophagus and stomach had significantly lower meridian energy in each of the 24 meridians. Though participants with abnormal duodenum findings had lower meridian energy, most of the differences did not reach statistical significance, except for the left small intestine meridian (28.91 versus 34.48, mean difference = −5.56, *P* < .01) and the left pericardium meridian (25.83 versus 29.89, mean difference = −4.05, *P* < .05).

## 4. Discussion

**Figure 4:**
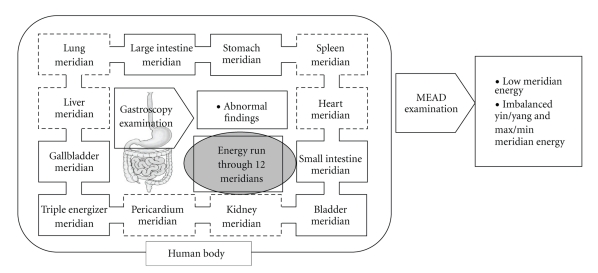
The proposed relationship between the abnormal gastroscopy findings and the meridian energy. Note that solid line represents yang meridians. Dotted line represents yin meridians.

 The results found that participants with abnormal stomach and esophageal findings using gastroscopy had significantly lower overall meridian energy and lower energy level in all 24 meridians (12 in the left and 12 in the right). Some may believe that abnormal stomach findings may be related more to the stomach meridian results and less to other meridian results, since the stomach meridian is more specifically connected to stomach organ than other meridians [1–5]. However, our results suggested a nonspecific relationship between abnormal gastric findings and meridian energy. The results supported the TCM theories that all meridians are interconnected and influence each other [1, 2, 4]. The lower energy levels among people with stomach and esophageal problems may suggest that these people are less able to preserve or produce meridian energy [1, 2]. The proposed relationship between the gastroscopy findings and the meridian energy is presented in Figure 4.

 Among the indicators for the balance of meridian energy, yin/yang and max/min ratios were different between people with normal and abnormal findings, and people with abnormal stomach findings who had a ratio value being farther from 1. The results suggest that people with abnormal stomach findings were more likely to have imbalanced meridian energy. The yin/yang and max/min ratios could be used as clinical indicators for imbalanced meridian energy. This study is unique in reporting associations between the structural abnormalities and meridian energy. More studies are needed to incorporate signs and symptoms in order to further examine the issue. Nonetheless, our results suggest that treatment of people with abnormal stomach findings should focus on enhancing meridian energy level and improving balance of meridian energy. 

 There were no significant differences in the overall mean meridian energy between subjects with and without abnormal findings in duodenum. Among the 24 meridians, subjects with abnormal findings in the duodenum showed significantly lower energy in the left small intestine meridian and the left pericardium meridian, but no significant differences in the other 22 meridians. The reason the structural abnormality in the duodenum did not relate to meridian energy at the same level as that in the stomach and esophagus merits further studies. 

### 4.1. Limitation

 Not all of our participants received gastroscopy, and we assumed that people who did not accept gastroscopy did not have abnormal findings. We believe that this approach was reasonable because if the patients had gastric symptoms they would have received gastroscopy. This belief was supported by our results that only 29 out of the 665 participants who received gastroscopy had normal findings. Ideally, we should have compared meridian energy among three groups, those with abnormal gastroscopy findings, those with normal gastroscopy findings, and those who had no gastroscopy. However only 29 participants had normal gastroscopy findings and the large variability in the energy data prohibited treating these 29 participants as a single group. Therefore, we combined participants who did not receive gastroscopy with participants who received gastroscopy and had normal findings to form the comparison group. Even if there was misclassification, the misclassification pointed to the direction of decreased and nonsignificant group differences. Therefore our results could be viewed as a conservative approach. This study was exploratory in nature and did not adjust for confounders. Though data were not shown, our findings of group differences sustained when participants with chronic illnesses were excluded. In addition, this study applied a cross-sectional design, therefore causal relationships could not be established. Further studies about the mechanisms linking abnormal gastroscopy findings and meridian energy results are needed.

## 5. Conclusions

 As energy medicine is used increasingly in patient treatment, health professionals could benefit from the knowledge of related alternative treatment. This study demonstrated that structural abnormalities in the esophagus and gastric areas were related to lower meridian energy. Whether enhancing meridian energy could improve gastric and esophageal health merits further studies [23, 24].

## Figures and Tables

**Table 1:** Characteristics of the study participants (*n* = 1,223).

Variable	*n*	(%)
Gender		
male	682	(55.8)
female	541	(44.2)
Age (years)		
<50	694	(56.7)
≥50	529	(43.3)
Heart disease		
No	1201	(98.2)
Yes	22	(1.8)
Diabetes		
No	1182	(96.6)
Yes	41	(3.4)
Hypertension		
No	1101	(90.0)
Yes	122	(10.0)
Hepatitis		
No	1100	(89.9)
Yes	123	(10.1)
